# Supplementary material for: Clinical test responses to different orthoptic exercise regimes in typical young adults
Source: Ophthalmic Physiol Opt. 2014 Jan 29;34(2):250–62. doi: 10.1111/opo.12109 (PMC4238796; doi:10.1111/opo.12109)
Supplement: Supplementary file 1 — Data S1. Testing protocol. [file 44402_2014_3402011_MOESM1_ESM.docx]

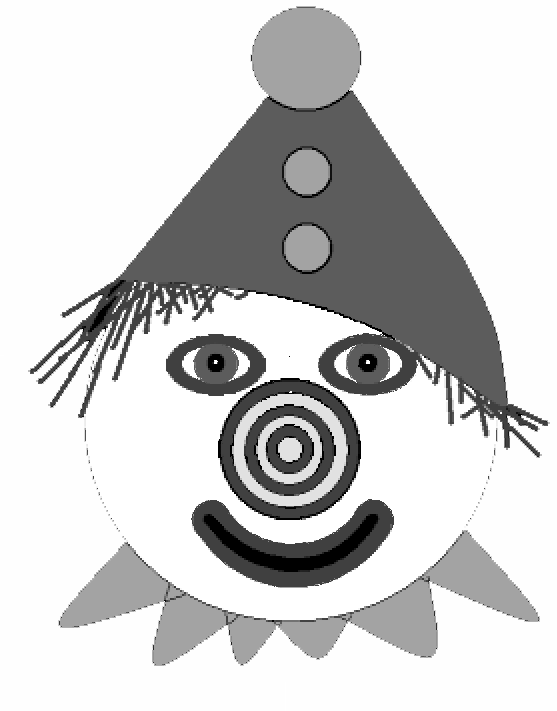


**Testing protocol**

**All Treatment Trial Participants EXCEPT the “no treatment” “Effort” group**

**History**

Any treatment as a child under 8?

Glasses/CLs – when 1^st^ used, Rx, how often worn

Orthoptic exercises – what, when, ?successful

Occlusion - successful

Surgery

When last refracted

**Augmented Convergence Insufficiency Symptom Survey (CISS)**

The Convergence Insufficiency Symptom Survey should be administered first before any other testing is administered.

Participant asked to fill in the questionnaire either before coming to the lab or before any other testing, explaining that this is a clinical score card used for people with eye strain, which we are using to confirm that they are within normal ranges.

The investigator should never give examples regarding any of the questions or response options.

**Corrected Visual Acuity (Distance and Near)**

If corrected visual acuity is 20/30 or worse in either eye, a subjective refraction should be

performed and visual acuity testing should be repeated with the best correction in a trial

frame.

**Refraction**

Test VA with a pinhole and refer to optometrists if VA reduced below 0.2 and cannot be corrected with simple additional minus. (Test in lab with this if undercorrected myope)

**Cover Test**

Near & Distance with glasses (and without if likely to be significant i.e. if hypermetropic)

**All subsequent tests to be carried out in a standard, friendly, positive, but even tone of voice with no stress on excessive effort although they should be told to report when they cannot keep things single or clear any more. Participants not specifically asked to try their very best and although they can be questioned during tests, should not be given extra instructions to “try harder”.**

**Test NPC, PFR, Vergence Facility, Accom Nr Pt , Accom Amplitude ONCE only after showing participant what is required and being sure they understand**

**Near Point of Convergence (NPC)**

Begin testing with subject wearing his/her optical Rx.

Use RAF Rule with the line & dot target.

Instruct subject “**Watch the target carefully, it might go blurry and it will eventually go double. Tell me when you can’t keep the target clear and then single any longer**.”

Slowly (1-2cm/sec) move target toward subject. When diplopia is reported stop moving

the target and ask the subject “Does it stay two or does it come back into one?” (NB “**come back into one”** not **“try to make it one”) !)**

If it comes back into one within 1-2 seconds, continue slowly moving the target

towards the patient until the patient is unable to regain fusion. Do not hold the

target in place for longer than 2 seconds.

If it stays double, this endpoint is the NPC break.

If the examiner observes a loss of fusion (without a report of double), the point at

which the examiner observed a loss of fusion is considered the NPC break.

If the patient continues to converge until the target is against the nose/brow (i.e.

break does not occur), measure how closely the subject converged and consider

this the NPC break.

Measure the NPC break to the nearest half centimetre (using the centre of the subject’s

forehead just above the level of the brow as the zero measure point from which the NPC

is taken.)

If the subject did not break, have the patient close or cover one eye for 3-5 seconds to

break fusion so that recovery can be measured.

Ask the subject to tell you “when it comes back together into one” (**NB again “comes back” not “make it come back”**) and slowly move the target away from the subject until the subject reports single vision or the examiner observes a recovery of fusion. This is the NPC recovery.

Measure the NPC recovery to the nearest half centimetre.

Measure the break and recovery as described.

between paired break/recovery measurements.

**TNO Stereotest at 40cm**

If SV >60secs then also do Randot

**Negative Fusional Vergence (NFV) at Near**

Test once unless unsure that participant understands, if so, show what you mean with weak prisms before doing full test.

Subject wearing his/her optical Rx.

Place target (Fixation Card Stick with single column of 20/30 letters) in primary gaze

33cm from subject’s eyes.

Ask the subject to “**tell me when the letters become blurred or become double (split into**

**2), but try to keep the target single as long as possible**” as BI prism is introduced.

Increase magnitude of BI prism at approx 2Δ/sec, pausing at each prism to confirm that

the target is “single and clear.”

If the subject reports blur, pause and note BI prism amount then continue to increase BI

prism pausing at each prism to confirm that the target is “single.” When the subject

reports double or break, ask subject ”Does it stay two or does it **come back into one**?”

Continue to introduce BI prism if subject recovers single vision. When subject can no

longer maintain single vision and has diplopia, note the BI prism amount and record this

value at the “BI break.”

After the subject reports diplopia, increase the BI prism by 5Δ, and then at a rate of about

2Δ /second, reduce the BI prism until the subject reports single vision. Consider this the

“recovery” finding. If recovery finding is higher than the break, the examiner should

repeat the entire measurement (blur, break and recovery).

If blur is not reported, record “X” on the data sheet (do not leave it blank).

NOTE: If diplopia is not reported but examiner notes loss of fusion, the prism through which

fusion is lost will be recorded as the “break” finding. Likewise, an examiner observation or

recovery of fusion will be recorded as “recovery.”

**Positive Fusional Vergence (PFV) at Near**

The examiner should wait 30 seconds after the NFV measure before performing

the PFV measures.

Test BO range as above but with a loose 20Δ prism before one eye (as long as have a range >20BO) and a prism bar before the other so that max range is 65Δ

NOTE: If the patient is able to fuse the largest (65Δ) prism, record 70Δ for the break value

and have the patient close or cover one eye to break fusion so that recovery can be measured.

Record the amount of prism through which the patient was able to regain fusion (maximum

value would be 45Δ

**Negative & Positive Fusional Vergence (PFV) at Distance**

As above but at 6m.

**Vergence Facility**

Use 12ΔBO / 3ΔBI flipper at 40cm fixing Policeman on Snellen Stick i.e. clarity not stressed.

Place plus side of 3ΔBI flipper before subject’s eyes. Ask subject to try to

make picture single as quickly as possible.

Instruct subject to report BV (say “one”) as soon as the letters are single.

Quickly flip the flipper to the 12ΔBOminus side, again instructing subject to report when single.

Prepare to begin timing for one minute using a stopwatch.

Start timing as you place the BI side of the flipper prisms in front of the subject’s eye.

Continue to alternate sides of flipper prisms for 1 minute, while counting the number of

“flips” of the prisms that the subject was able to clear. A flip = cycle of overcoming both prisms.

**Ocular Movements**

To exclude incomitance and to allow a break for natural binocular vision at intermediate distance.

**Binocular Near Point of Accommodation**

Subject wearing his/her optical Rx

Use RAF Rule with N5 patch of text

Instruct the subject to, “**Tell me when the letters first start to blur, but try to keep the**

**letters clear as long as possible.**”

Slowly move the target toward the subject at approximately 1 to 2 cm/sec until subject

reports first blur. Ask if the letters stay blurry or become clear. If target becomes clear,

continue moving target closer until blurred. Stop at “first sustained blur.”

Measure to the nearest one-half centimeter (using forehead just above the level of the

subject’s brow as the zero measure point).

**Monocular Amplitude of Accommodation (only test one eye for this study)**

As above but cover the non-dominant eye – let participant choose.

**Binocular Accommodative Facility (test once)**

Participant wearing his/her optical Rx.

Use Accommodation Fixation Stick at 40 cm distance.

Show the participant what will be involved (no more than 2 repetitions)

Place plus side of +/- 2.00 lens flipper before subject’s eyes. Ask subject to try to

make letters clear as quickly as possible.

Instruct subject to report clarity (say “clear”) as soon as the letters are clear.

When letters are reported to be clear, quickly flip the flipper to the minus side, again

instructing subject to read letters & report when clear.

Prepare to begin timing for one minute using a stopwatch.

Start timing as you place the plus side of the flipper lens in front of the subject’s eye.

Continue to alternate sides of flipper lenses for 1 minute, while counting the number of

“flips” of the lens that the subject was able to clear. A flip = cycle of overcoming both +&- lens

NOTE: Even if the subject has difficulty (i.e., is slow or takes a while) clearing a lens, testing

should be continued for a full minute. However, if the subject cannot clear one side of the

flipper lens in one minute, then 0 flips will be recorded but repeat, trying, say -2.00/∞ or +2.00/∞.

NOTE: The lenses should be “flipped” from one side to another, not slid/moved up and down in

front of the subject’s eye.

Allow at least a minute break before proceeding to the next test

**Uniocular Accommodative Facility (test once)**

As above but with one eye occluded (participant can choose)

**Prism CoverTest**

Nr & Distance, with & without Rx

**In the Lab**

Tell the participant to “just look at the targets” presented. Tell them to ignore the faint infra-red camera dots if they notice them

**“No Treatment - Effort” group**

Do all tests as before, but this time really emphasise the effort they should be making to get the maximum possible response. Encourage them throughout with “go on”, “try a bit harder”, you’re doing really well”, “let’s see if you can do a bit more”, “don’t worry if it pulls a bit, try a bit more” etc etc. Be enthusiastic and “over the top”.

**In the Lab**

This time really encourage them to concentrate on getting the images as clear and joined as possible. Look at, and concentrate on, the rings on the clown’s nose.
