# Supplementary material for: Clinical test responses to different orthoptic exercise regimes in typical young adults
Source: Ophthalmic Physiol Opt. 2014 Jan 29;34(2):250–62. doi: 10.1111/opo.12109 (PMC4238796; doi:10.1111/opo.12109)
Supplement: Supplementary file 1 [file opo0034-0250-SD1.pdf]

OPO OPHTHALMIC & PHYSIOLOGICAL OPTICS
THE JOURNAL OF THE COLLEGE OF OPTOMETRISTS

Ophthalmic & Physiological Optics ISSN 0275-5408

Clinical test responses to different orthoptic exercise regimes in typical young adults

Anna Horwood and Sonia Toor

School of Psychology & Clinical Language Sciences, University of Reading, Reading, UK

Citation information: Horwood A & Toor S. Clinical test responses to different orthoptic exercise regimes in typical young adults. *Ophthalmic Physiol Opt* 2014; **34**: 250–262. doi: 10.1111/opo.12109

Keywords: accommodation, convergence, fusion, orthoptic exercises, vision therapy

Correspondence: Anna Horwood
E-mail address: a.m.horwood@reading.ac.uk

Received: 15 August 2013; Accepted: 13 November 2013; Published Online: 29 January 2014

Abstract

Purpose: The relative efficiency of different eye exercise regimes is unclear, and in particular the influences of practice, placebo and the amount of effort required are rarely considered. This study measured conventional clinical measures following different regimes in typical young adults.
Methods: A total of 156 asymptomatic young adults were directed to carry out eye exercises three times daily for 2 weeks. Exercises were directed at improving blur responses (accommodation), disparity responses (convergence), both in a naturalistic relationship, convergence in excess of accommodation, accommodation in excess of convergence, and a placebo regime. They were compared to two control groups, neither of which were given exercises, but the second of which were asked to make maximum effort during the second testing.
Results: Instruction set and participant effort were more effective than many exercises. Convergence exercises independent of accommodation were the most effective treatment, followed by accommodation exercises, and both regimes resulted in changes in both vergence and accommodation test responses. Exercises targeting convergence and accommodation working together were less effective than those where they were separated. Accommodation measures were prone to large instruction/effort effects and monocular accommodation facility was subject to large practice effects.
Conclusions: Separating convergence and accommodation exercises seemed more effective than exercising both systems concurrently and suggests that stimulation of accommodation and convergence may act in an additive fashion to aid responses. Instruction/effort effects are large and should be carefully controlled if claims for the efficacy of any exercise regime are to be made.

Introduction

Orthoptic exercises have been an established part of therapy for heterophoria, intermittent strabismus, convergence insufficiency and accommodative problems for many years, but their comparative effects have not been comprehensively reported. A major review by Barrett[1] concluded that although there is some evidence that exercises are effective for some conditions such as convergence and accommodation anomalies, the research is still incomplete and attention and placebo effects are often unquantified. Even carefully designed and validated studies such as the Convergence Insufficiency Treatment Trials[2,3] where exercises did appear effective found significant improvements in symptoms and clinical measures after placebo treatments, so this is clearly a problem when assessing exercise efficacy in both group studies and on an individual level.

A fundamental tenet of orthoptic exercises is that although convergence and accommodation may be trained, exercising *relative* vergence or accommodation is also necessary and will achieve the best and most long lasting results. This principle is found throughout both optometric and orthoptic clinical textbooks.[4–6] Despite this widely held consensus among the optometry and orthoptic professions,

© 2014 The Authors. Ophthalmic & Physiological Optics published by John Wiley & Sons Ltd on behalf of The College of Optometrists.

This is an open access article under the terms of the Creative Commons Attribution License, which permits use, distribution and reproduction in any medium, provided the original work is properly cited.

objective assessment of their effects is rare, although some recent studies have addressed the issue.[7,8]

Maxwell *et al*[8] suggest that volitional vergence may mediate changes in accommodation velocity after training, and results from our laboratory and others also suggest a primary role for disparity as a major drive to both vergence and accommodation in naturalistic situations where a range of cues are available.[9,10] Although these studies suggest that exercising convergence would also not only help convergence, but also accommodation via the CA/C linkage, the pervading impression is that the vergence induced by accommodation (AC/A) is equally, or more influential, i.e. using accommodation to change the angle, for example in exodeviations,[11,12] rather than convergence to change the angle and accommodation.

Rouse *et al*[13,14] have addressed practice effects on monocular and binocular accommodative facility and a review[15] pinpointed the variability of these tests and the necessity for careful control of clinical and experimental factors when assessing efficacy. Others have attempted to address instruction set[16–18] and have shown that it can be influential in affecting responses, but it is still not clear how different traditional exercise modalities fit into more recent thinking, or how important practice, placebo and patient/tester interaction effects are in comparison to true treatment effects. Despite many papers reporting subjective and clinical improvements after vision therapy, there are fewer studies that compare response to different exercise modalities in similar participants, which assess multiple measures made under standard conditions, or which make objective measurements.

As a precursor to studying patient groups, we have been studying the effect of eight different "treatment" regimes on objective measures of accommodation and convergence[19] taken from naïve typical young adults to establish baseline measures. In the course of this objective study we also collected a large dataset of conventional clinical measures of vergence and accommodation function in response to treatment regimes in closely matched groups. This paper reports these clinical results.

Methods

The study protocol adhered to the Declaration of Helsinki and was scrutinised and approved by the University of Reading Research Ethics Committee. Participants were recruited from the School of Psychology Research Participants Database and by advertisements within the University. All were undergraduates or post-graduates between 18 and 25 years of age studying psychology or other sciences. Participants were ineligible if they had a strabismus, had any history of seeking treatment for binocular vision problems, or had taken part in any visual experiment before. A primary selection criterion was that they "considered themselves to have normal eyes", although mild corrected refractive errors (up to ±4.00 DS) were accepted. They were told that the study was comparing the results of exercises that targeted different aspects of the visual system in comparison to a no treatment group. They were rewarded with either course credit to be redeemed when carrying out their own unrelated research, or were paid a small fee for their time if they were recruited from outside the School.

Initial visit

Before the visit the participants completed the Convergence Insufficiency Symptom Survey[20,21] so that we could identify and exclude those with any significant visual symptoms. We adjusted the scores downwards for some of the test items e.g. feeling sleepy when doing close work, or having to re-read words, to account for a student lifestyle.[19] On their first visit to the laboratory a brief history was taken to ensure no history of binocular vision problems and to verify that any refractive correction had been checked within the last year. All testing was carried out using their habitual spectacles or contact lenses. The testing room was artificially lit (350 lux) so was not affected by varying light levels on different days.

The main purpose of the study was to assess objective convergence and accommodation responses using a Plusoptix PowerRefII autorefractor.[9,19] The participants were asked to watch a range of stimuli moving between 25 cm and 2 m in a testing session which lasted about 7 min.

After this session, a qualified orthoptist (ST) carried out baseline measures of visual status. For all near fixation tasks we used a vertical column of N5 letters as the target and asked the participants to fixate single letters, keeping them clear, to try to control accommodative demand as much as possible. Orthoptic testing assessed corrected monocular logMAR visual acuity using a ETDRS chart, cover test, ocular motility assessment, stereoacuity using the TNO stereotest, objective convergence near point to the accommodative target (NPC), monocular and binocular accommodative near point (MNPA and BNPA) (all using a RAF Near Point Rule using push-up methods), base out (BO) and base in (BI) prism fusion range (PFR) to blur, diplopia and recovery at 33 cm (N) and 6 m (D), monocular and binocular accommodative facility (MAF and BAF) at 33 cm using ±2D flipper lenses and vergence facility (VF) using 12ΔBO/3ΔBI flipper prisms (recorded in "flip cycles" per minute, cpm) and alternate prism cover test at 33 cm and 6 m. Monocular tests were carried out using the preferred eye. All the different facility tests were carried out over one-minute periods and were separated by a few minutes of natural binocularity of between the tests. Before

© 2014 The Authors. Ophthalmic & Physiological Optics published by John Wiley & Sons Ltd on behalf of The College of Optometrists.

each timed testing period, the participant was shown what effect the lenses/prisms had, were given a brief practice period (no more than two repetitions) and the examiner satisfied herself that they understood and were able to do what they were expected to do. We did not check for suppression in the binocular accommodation facility test, but did check objective eye movements for the VF test. The extreme dissociation of the prism cover testing was only carried out at the end of testing. Testing was carried out in the same order for all participants and on each of the two visits to the laboratory because studies have shown that test order can cause significant differences.[22] We took extreme care to use a standard testing protocol modelled on that used by the Convergence Insufficiency Treatment Trial group[20] (see Data S1). The tester used a friendly, positive tone of voice, with wording such as "Watch the target carefully, it might go blurry and it will eventually go double. Tell me when you can't keep the target clear and then single any longer". Each test was carried out only once on both visits, but only when it was clear that the participant understood what was required of them.

A further autorefractor recording session was then carried out before the participants were taken to another room to be allocated to a treatment group by a second experimenter (AH) masked to any laboratory results.

Participants were randomised to a treatment group using a random number generator. They were told which aspect of their vision the exercises were targeting, were shown how to do them and were then asked to demonstrate them back to the experimenter. We tried to match exercise regimes for difficulty, type and number of different tasks. All exercise groups carried out three different tasks every session, involving both near and distant fixation and including slow/gradual effort, a "to nose" task, and rapid "jump" tasks (*Table 1*) and if time permitted they were told to concentrate on any task they found particularly challenging and to try and improve on past scores.

Participants were asked to carry out the exercises for 5 min, three times a day for 2 weeks. To maximise adherence to the protocol, they were asked to set their mobile phone alarms to remind them to do them regularly. The participants were all science students so were reminded of the importance of honest reporting of any missed sessions in relation to experimental accuracy, and were also given a diary sheet to fill in to record near points or flipper task scores. They were told that we expected to be able to relate

Table 1. Details of exercise regimes

Group	Skill manipulated	Target	Exercise	Subj End point
Blur	Accommodation only. Blur independent of disparity.	N5 letters/distance details e.g. text or tree leaves	Monocular push-ups (near to nose)<br>Monocular near/distance "jump" accommodation (near/distance)<br>Monocular accommodation facility (+2/−2D (near) 0/−2D (distance) lens flippers)	Blur
Both	Accommodation & convergence in normal relationship	N5 letters/distance details	Binocular push-ups (near to nose)<br>Binocular "jump" vergence/accommodation (near/distance)<br>Near/distance physiological diplopia	Blur *or* Diplopia
Disparity	Vergence independent of accommodation	Gabor image/building/ clouds	Binocular push-ups (near to nose)<br>Binocular "jump" vergence (near/distance)<br>Near & distance vergence facility (12ΔBO/4ΔBI prism flippers)	Diplopia
Convergence +	Convergence in excess of accommodation	N5 letters/distance details	Binocular push-ups (+2.0D or 12ΔBO) (near to nose)<br>Binocular near accommodation facility(0/+2.0D)<br>Binocular near & distance vergence facility (0/12ΔBO)	Blur *or* Diplopia
Accommodation +	Accommodation in excess of convergence	N5 letters/distance details	Binocular push-ups (−2.0D or 12ΔBI) (near to nose)<br>Binocular near & distance accommodation facility (0/−2.0D)<br>Binocular near (& distance if possible) vergence facility (0/12ΔBI)	Blur *or* Diplopia
Motion (placebo)	Attention, motion detection, proprioception	Visual illusions. Physical objects	"Snakes illusion" – max/min moving (near)<br>Necker cube – perceptual shift (near and distance)<br>Yoked prisms – visually directed reach with/without prisms (near and walking towards and touching distance target)	
Nil	Practice, test/retest		None	
Effort	Tester, instruction set, effort		None	

© 2014 The Authors. Ophthalmic & Physiological Optics published by John Wiley & Sons Ltd on behalf of The College of Optometrists.

laboratory results to these diary records, so we would probably be able to tell if they had missed many homework sessions.

Exercise groups

1. Blur. These exercises concentrated on accommodation to resolve blur induced by near text or lenses, independent from vergence. They were necessarily monocular (each eye practiced in turn) because convergence to resolve disparity can drive a large proportion of the accommodation response.[9,10,23] The participants were asked to concentrate on maintaining maximum clarity of a detailed target (similar to 5 point text or smaller) at all times. The end point of any exercise was when clarity could no longer be maintained as blur was induced by both target motion (push-up/more distance fixation, "jump" near distance fixation) and flipper lenses (+2.00/-2.00).
2. Both. Accommodation and convergence typically act together, so these exercises were carried out binocularly, stressing clarity *and* single vision at all times using a detailed target and slow push-up and "jump"/facility tasks for near and distance. The end point of any exercise was when either blur *or* diplopia could not be prevented. They also practiced appreciating and manipulating physiological diplopia, paying attention to relative blur and doubling of images compared to the clear, single fixation plane.
3. Disparity. The participants were given a fixation card with a printed blurry "Gabor patch" target set against a grey background, which contained fusible elements, but which looked subjectively similar when the image was optically blurred and so would induce minimal accommodation. Blur cues could thus be minimised as a drive to convergence via the AC/A linkage, and vergence-induced accommodation would not be noticed. This fixation target was to be used for all near tasks, and a large distant fixation target, such as a building, cloud or tree was used for distance fixation, and the participants were told that it did not matter if it blurred as long as it was single. They carried out gradual and "jump" convergence/divergence tasks between near and distance fixation.
4. Con+. Many orthoptic exercises for exodeviations ask for convergence to be used in excess of accommodation for a given distance, or for accommodation to be relaxed in relation to convergence. The participants were given a set of flippers containing a pair of +2.0D lenses right and left, and pair of 12Δ BO prisms (6Δ each eye). Practice involved maintaining clear and single vision as lenses/prisms were introduced and then removed and also gradual push-ups/relaxation through the lenses/prisms. When looking through the lenses at any target vergence would be appropriate but with less accommodation required. The lenses were only used for practice at near fixation to avoid insuperable distance blur. When looking through the prisms at any target, appropriate accommodation but additional convergence would be required. A detailed fixation target for near (5 point letters) and far distance (resolving leaves on a tree or text on a sign out of a window) was used and the importance of both clarity and single vision were stressed at all times.
5. Acc+. Participants were given a set of flippers containing a pair of −2.0D lenses and a set of 12Δ BI prisms (6Δ each eye). When looking through the lenses more accommodation, but normal vergence, would be required, and when looking through the prisms divergence (or less convergence) would be required for a normal amount of accommodation for the target distance. The detailed accommodation target was again used as in the above group, stressing clarity and single vision while doing similar tasks to the Con+ group for both near and distant fixation.
6. Motion. For this placebo treatment the participants were told that these exercises were targeting motion detection, the position of images in space and proprioception. They involved using two different optical illusions (a Necker cube, and the Snakes illusion) to practice making perceptual shifts, and yoked base right or base up prisms while doing reaching tasks, to alter proprioceptive/visual input. Blur/clarity or diplopia/single vision were not mentioned.
7. Nil. The participants were told they were in a control group looking at repetition effects, so did not need to do any exercises, but just return for repeat testing. For this and all the above groups, the tester was masked to treatment allocation.
8. Effort. This group was also told they were in the no-treatment control group and did not need to do exercises, but this was the only group to which the tester was not masked to treatment allocation for the second visit. On this visit the tester emphasised the use of effort and concentration on all the laboratory and clinical tests, encouraging them to "try harder" and "really concentrate" throughout.

The participants returned 2 weeks later for repeat testing with the tester masked to previous results and treatment allocation.

Statistical analysis

Data were entered on a spreadsheet and initially analysed with Excel. Further analysis was carried out using SPSS 18 using mixed ANOVA with pre-/post-treatment change as a

© 2014 The Authors. Ophthalmic & Physiological Optics published by John Wiley & Sons Ltd on behalf of The College of Optometrists.

within-groups factor and exercise group as a between groups factor and alpha levels of 0.05. All responses were assessed for normality of distribution by assessing z-scores of skew and kurtosis and data transformed if values exceeded 1.96. Post hoc testing used paired t-tests. Because multiple comparisons were made across the dataset we used a more conservative alpha level of 0.01 to reduce the likelihood of Type I error, while avoiding excessive Type II error risked by Bonferroni correction.

Results

Of the 172 participants recruited, 14 were excluded because they showed evidence of CI according to the Convergence Insufficiency Treatment Trial group criterion (an adjusted Convergence Insufficiency Symptom Survey score of ≥20,[21], a previously undiagnosed strabismus, or mild accommodative spasm (despite all considering their eyes normal). Fifteen had an unadjusted Convergence Insufficiency Symptom Survey score of >20 which reduced to <20 on adjustment and they were included in the analysis. We took particular care to ensure they were sure their symptoms were due to lifestyle issues (working when very tired, being dyslexic or doing complex reading that they struggled to understand), and that their orthoptic measures were well within normal limits. Two further participants were excluded due to unusual eyelash and lid configuration which made it difficult to obtain enough accurate photorefraction data for the other arm of the study. 98% of the participants returned exactly 2 weeks later at the same time of day and of the seven that did not (equally spread across the groups), only two came at a different time and the others either 1 or 2 days later than 14 days.

Two participants admitted by email before the second test that they had not bothered to do the exercises at all and so they were allocated to one of the "no treatment" groups, and the tester remained masked to initial group allocation. Data from 156 participants were analysed, and each group contained at least 17 participants. At the debrief after the experiment had been completed, only one of the 21 Motion group participants had suspected they had been in a placebo group.

There were no significant differences between the groups in terms of mean spherical refractive error ($F_{7,148} = 0.73$, $p = 0.65$), initial heterophoria at near ($F_{7,146} = 1.84$, $p = 0.08$) or at distance ($F_{7,141} = 1.32$, $p = 0.24$). Differences in orthoptic measures at baseline were small and non-systematic between the groups. Twenty-five (16%) participants, numbering two to six in each group, were unable to clear the +2.0D lens of the lens flippers when monocular accommodative facility was tested on the first visit. If this was the case a plano/−2.0D alternation was used instead for both testing sessions. Between groups ANOVA of the baseline orthoptic measures showed small but significant main effects of group for VF ($F_{7,145} = 2.49$, $p = 0.019$), Near BO fusion range to diplopia ($F_{7,147} = 2.1$, $p = 0.047$), Near BI fusion range to diplopia ($F_{7,147} = 2.61$, $p = 0.015$) and Near BI fusion range recovery ($F_{7,148} = 2.173$, $p = 0.04$). The only significant post-hoc differences between the groups at baseline were in the Near BI Fusion range to diplopia, where the Con+ group had a larger initial range (18.3Δ) than both the Blur (13.9Δ) and the Nil groups (13.8Δ).

Analysis by treatment group

For some of the measures e.g. convergence and accommodation near points, performances were at or near ceiling before treatment, with only limited scope for improvement, but it was clear that some exercise regimes produced greater improvements than others. All groups except the Nil group improved somewhat, but in many cases these improvements did not reach statistical significance.

Table 2 illustrates the changes we considered significant ($p < 0.01$) or marginal ($p = 0.01–0.05$). The Nil responses remained very similar for most measures, but deteriorated very slightly (non-significantly) for all fusion ranges. As the different tests used different measurement scales and different typical ranges, we initially calculated percentage change across each of these the different measures by treatment group to obtain a broad overview.

Of the different exercise regimes, the Disparity group made the greatest overall improvement (by 17.2% averaged across the different measures), closely followed by the Blur group (16.1%), and both groups showed increases of more than 20% on 7 of the 14 measures, with BO fusion ranges improving the most. In both of these groups, improvements occurred not only in the visual skill (vergence or accommodation) that had been exercised, but more widely e.g. monocular accommodation exercises improved fusion ranges, and vergence exercises independent of detail detection improved binocular accommodation facility.

The greatest overall change, however, was in the Effort group, who improved their responses by 27% across the different measures and made greater than 20% improvement in nine of the 14 measures, so it appears that the effect of just stressing additional effort was more effective than any exercise regime.

It was notable that treatment regimes concentrating on accommodation and convergence being exercised simultaneously and in relation to each other had lesser effects than when they were exercised separately.

Monocular accommodation facility increased dramatically on the second visit in all groups, including both control groups, suggesting it is subject to large practice effects.

© 2014 The Authors. Ophthalmic & Physiological Optics published by John Wiley & Sons Ltd on behalf of The College of Optometrists.

Table 2. Extent and significance of statistically significant change (improvements) in clinical measures

	**Treatment Group**							
**Test**	**Blur**	**Both**	**Disparity**	**Con+**	**Acc+**	**Motion**	**Nil**	**Effort**
NPC	1.5 cm<br>($p = 0.003$)		1.5 cm<br>($p = 0.01$)					2 cm<br>($p = 0.0002$)
VF		2.4 cpm<br>($p = 0.0004$)	4.75 cpm<br>($p = 0.0002$)		2.46 cpm<br>($p = 0.02$)	2.0 cpm<br>($p = 0.01$)		3.6 cpm<br>($p = 0.001$)
BNPA				1.05 cm<br>($p = 0.04$)				0.95 cm<br>($p = 0.03$)
MNPA			0.8 cm<br>($p = 0.008$)	0.8 cm<br>($p = 0.04$)				1.57 cm<br>($p = 0.002$)
BAF	1.90 cpm<br>($p = 0.04$)	2.33 cpm<br>($p = 0.03$)			1.56<br>($p = 0.04$)			3.04 cpm<br>($p = 0.003$)
MAF	5.02 cpm<br>($p = 0.005$)	3.0 cpm<br>($p = 0.004$)		3.55 cpm<br>($p < 0.0001$)	3.3 cpm<br>($p = 0.02$)	3.8 cpm<br>($p = 0.04$)	3.05 cpm<br>($p = 0.02$)	2.57 cpm<br>($p = 0.03$)
NBOD	9.3 PD<br>($p = 0.02$)		10.05 PD<br>($p = 0.02$)					9.76 PD<br>($p = 0.003$)
NBOR	10.05 PD<br>($p = 0.02$)		9.55 PD<br>($p = 0.05$)					7.7 PD<br>($p = 0.03$)
DBOD			9.75 PD<br>($p = 0.001$)	4.8 PD<br>($p = 0.04$)				6.09 PD<br>($p = 0.03$)
DBOR			7.05 PD<br>($p = 0.002$)	5.72 PD<br>($p = 0.004$)				
NBID					3.8 PD<br>($p = 0.004$)			2.42 PD<br>($p = 0.05$)
NBIR					2.37 PD<br>($p = 0.03$)			2.47 PD<br>($p = 0.04$)
DBID								
DBIR								

Shaded cells = $p < 0.01$ Blank cells $p > 0.05$ cpm, "flip cycles" per minute; NPC, near point of convergence; VF, vergence facility; BNPA, binocular near point of accommodation; BAF, binocular accommodation facility; MNPA, near point of accommodation; MAF, monocular accommodation facility; NBOD, near BO PFR diplopia point; NBOR, near BO PFR recovery point; NBID, near BI PFR diplopia point; NBIR, near BI PFR recovery point; DBOD, distance BO PFR diplopia point; DBOR, distance BO PFR recovery point; DBID, distance BI PFR diplopia point; DBIR, distance BI PFR recovery point.

Only 33% of the participants noticed blur points when PFRs were tested (range 12–68% across treatment groups and between first/second testing) and there were no consistent patterns of differences between the treatment groups. Although most people either did, or did not notice blur points, it was not even always consistent on the first and second visit, or between near and distance, or base in and base out in the same individual. Although instructed to identify blur points, and four of the groups had been explicitly practicing keeping targets clear, when asked if they had noticed blur after the test had finished they often commented that "I didn't notice", which could have meant that blur did not occur, or had occurred but had been ignored. In particular, doing exercises concentrating on clarity did not make these groups any more likely to notice blur points on the second test. Analysis of blur points was limited due to small numbers, with fewer than five participants noticing blur in the Effort and Blur groups for near fixation, and in all groups except the Nil group for distance fixation. There is also no objective check possible for this measure (unlike diplopia and recovery points where an experienced tester can see the eye movement). We therefore decided that these data were unreliable and any analysis would lack sufficient power, so they were not analysed further.

Analysis by test response

Convergence measures

Convergence near point (NPC). These data were positively skewed as many participants performed well, so analysis used log transformed values. Convergence near point improved overall (main effect of change $F_{1,148} = 26.83$, $p < 0.0001$), but with a significant interaction with group ($F_{7,148} = 2.42$, $p = 0.022$) (*Figure 1*). Post hoc testing showed that only the Blur ($p = 0.003$) group (where convergence had not been involved in the treatment), Disparity ($p = 0.01$), and Effort ($p = 0.0002$) groups improved significantly. The absence of effect in the non-treatment groups suggests that there may be a true treatment effect from most exercise regimes.

© 2014 The Authors. Ophthalmic & Physiological Optics published by John Wiley & Sons Ltd on behalf of The College of Optometrists.

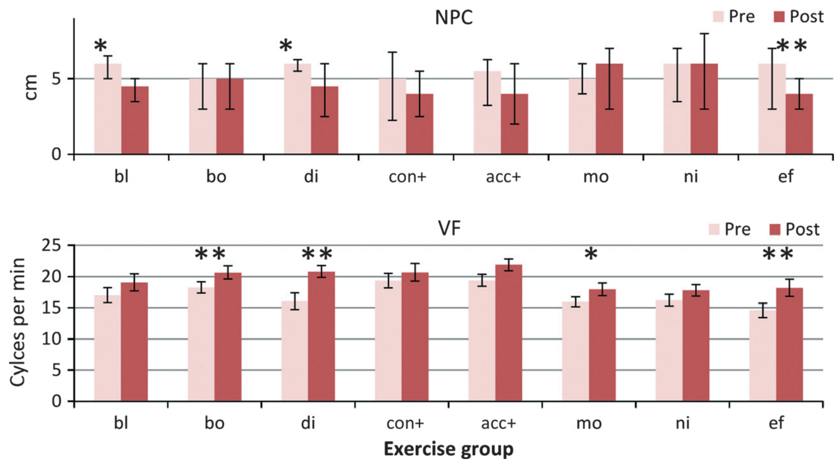

Figure 1. Convergence measures. Pre- and post-treatment convergence measures for each treatment group. Abbreviations: NPC, Near point of convergence (in cm.)(NB Median and inter-quartile range error bars for this measure as not-normally distributed) VF, near vergence facility using 12ΔBO/3ΔBI flipper prisms. Group abbreviations; bl, blur/accommodation treatment; bo, both (simultaneous convergence and accommodation treatment); di, disparity (convergence treatment); con+, convergence in excess of accommodation treatment; acc+, accommodation in excess of convergence treatment; mo, motion (placebo treatment); ni, nil (no treatment controls); ef, effort, no treatment. *$p < 0.01$; **$p < 0.001$.

Vergence facility (VF). There was a highly significant change in responses (main effect $F_{1,145} = 73.8$, $p < 0.00001$) and marginal group/change interaction ($F_{7,145} = 1.93$, $p = 0.07$). Vergence facility improved in the Both ($p = 0.0004$), Disparity ($p = 0.0002$), Motion ($p = 0.01$) and the Effort ($p = 0.001$) (*Figure 1*) groups. If the effect of just doing the test a second time (the mean improvement of the Nil group) is subtracted from the scores, there was a less marked overall change ($F_{1,127} = 7.05$, $p < 0.001$), and the only significant improvement over the baseline measures was in the Disparity group ($t_{19} = 2.96$, $p = 0.008$).

Accommodation measures

Binocular accommodation near point (BNPA). There was a small improvement in accommodation near points, with a significant main effect ($F_{1,148}$) = 10.85, $p = 0.001$) and no significant interaction (*Figure 2*). No individual post-hoc comparison reached our required alpha level of 0.01.

Monocular near point of accommodation (MNPA). There was a slight improvement overall (main effect: $F_{1,148} = 18.51$, $p < 0.0001$), but there was no significant interaction with group. Significant changes only occurred in the Disparity group ($p = 0.008$), (who had not been practicing accommodation, but who had practiced convergence) and also in Effort group ($p = 0.002$) (*Figure 2*).

Binocular accommodation facility (BAF). All groups improved their scores somewhat for binocular accommodation facility (main effect $F_{1,148} = 29.74$, $p < 0.00001$), but with no significant interaction. Significant improvements were found only in the Effort group ($p < 0.01$), although there were smaller changes in the Blur ($p = 0.04$), Both ($p = 0.03$), Acc+ ($p = 0.04$) groups, but if the improvement in the Nil group was subtracted from the scores, none of these changes approached significance.

Monocular accommodation facility (MAF). This was the test where most proportional improvement occurred, with a strongly significant main effect of change ($F_{1,145} = 55.33$, $p < 0.00001$) and no significant interaction between the groups. Even the Nil group improved their scores dramatically on the second visit from means of 7.3 to 10.4 cycles per minute. Many participants in all groups found this the most difficult test, with some only able to perform the test with a plano/−2.0D combination, and all except the Disparity group (where attention to clear vision had been explicitly excluded) improved significantly on second testing, with similar improvements in the treatment, placebo and no-treatment groups. The greatest changes were found in the Blur ($p = 0.005$) (where the task had been specifically practised), Both ($p = 0.0004$) and Con+ ($p < 0.0001$) groups (where it had not). If the mean change in the Nil group is subtracted from the scores, no improvement approached significance in any other group.

Prism fusion ranges

BO fusion ranges. All exercise groups except the Both group improved their near and distance BO fusion ranges somewhat to both diplopia and recovery (*Figure 3*) (main effects

© 2014 The Authors. Ophthalmic & Physiological Optics published by John Wiley & Sons Ltd on behalf of The College of Optometrists.

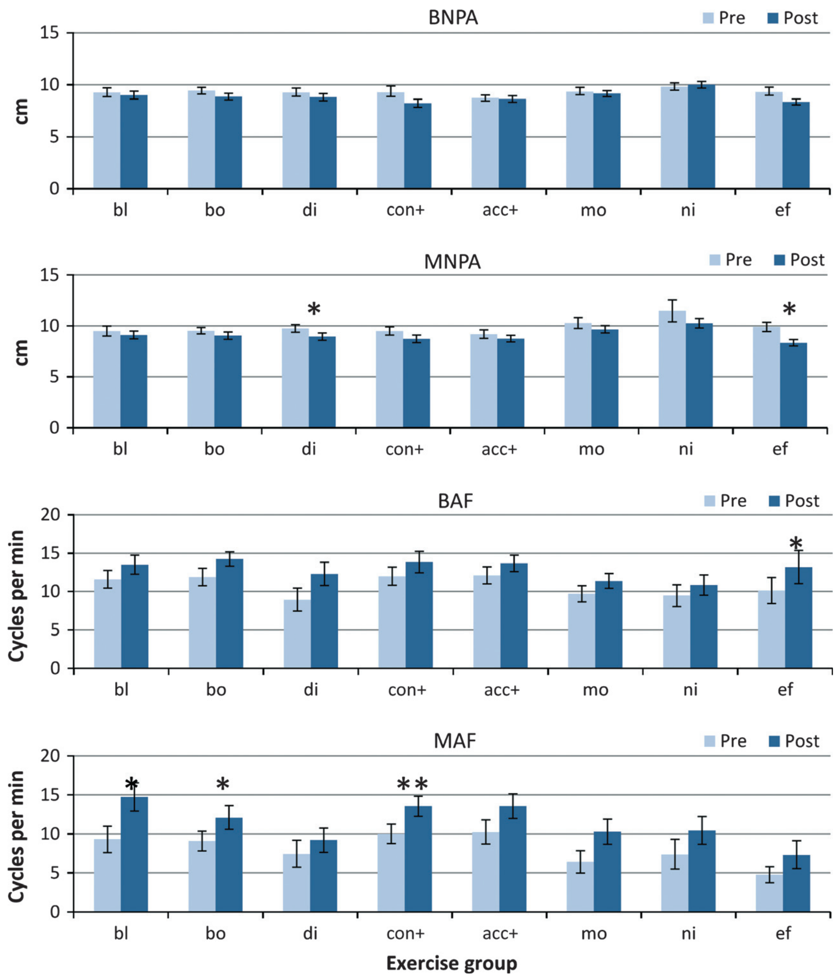

Figure 2. Accommodation measures. Pre- and post-treatment accommodation measures for each treatment group (Error bars: Standard error. BNPA, binocular near point of accommodation; MNPA, monocular near point of accommodation; BAF, near binocular accommodation facility; MAF, near monocular accommodation facility. Group abbreviations; bl, blur/accommodation treatment; bo, both (simultaneous convergence and accommodation treatment); di, disparity (convergence treatment); con+, convergence in excess of accommodation treatment; acc+, accommodation in excess of convergence treatment; mo, motion (placebo treatment); ni, nil (no treatment controls); ef, effort, no treatment. *$p < 0.01$; **$p < 0.001$.

at least $F_{1,148} > 10.25$, $p < 0.002$ in all cases, with significant group vs change interactions ($p < 0.05$) in all except the distance recovery point (which was marginal $p < 0.1$). The Nil and Motion groups did not improve significantly.

Specifically, the near BO ranges only improved significantly in the Effort group ($p = 0.003$ to diplopia and marginally $p = 0.05$ to recovery), and only improved marginally in the Blur, Disparity and Effort groups (Blur: $p = 0.02$ to diplopia and $p = 0.02$ for recovery; Disparity: $p = 0.02$ diplopia/$p = 0.05$ recovery) In the distance only the Disparity group improved ($p = 0.001$ diplopia/ $p = 0.002$ recovery), with marginal effects in the Effort group ($p = 0.03$ diplopia/$p = 0.07$ recovery). The Con+ group improved more at distance ($p = 0.04$ diplopia/ $p = 0.004$ recovery) than for near. There were no statistically significant differences in the Both, Acc+, Motion or Nil groups for any BO fusion range. This suggests that some true treatment effects may be present for BO fusion ranges.

Base in fusion ranges. Any changes in BI fusion range were very small (*Figure 4*) and only for near fixation (main effect of change $F_{1,147} = 10.74$, $p = 0.001$ and $F_{1,147} = 13.04$, $p < 0.001$) for diplopia and recovery points respectively). There was no significant main effect of change for distance fixation.

The only exercise group that showed any significant change was the Acc+ group for near fixation ($p = 0.004$ diplopia/$p = 0.04$ recovery), who had specifically been practising divergence in relation to accommodation. Effort

© 2014 The Authors. Ophthalmic & Physiological Optics published by John Wiley & Sons Ltd on behalf of The College of Optometrists.

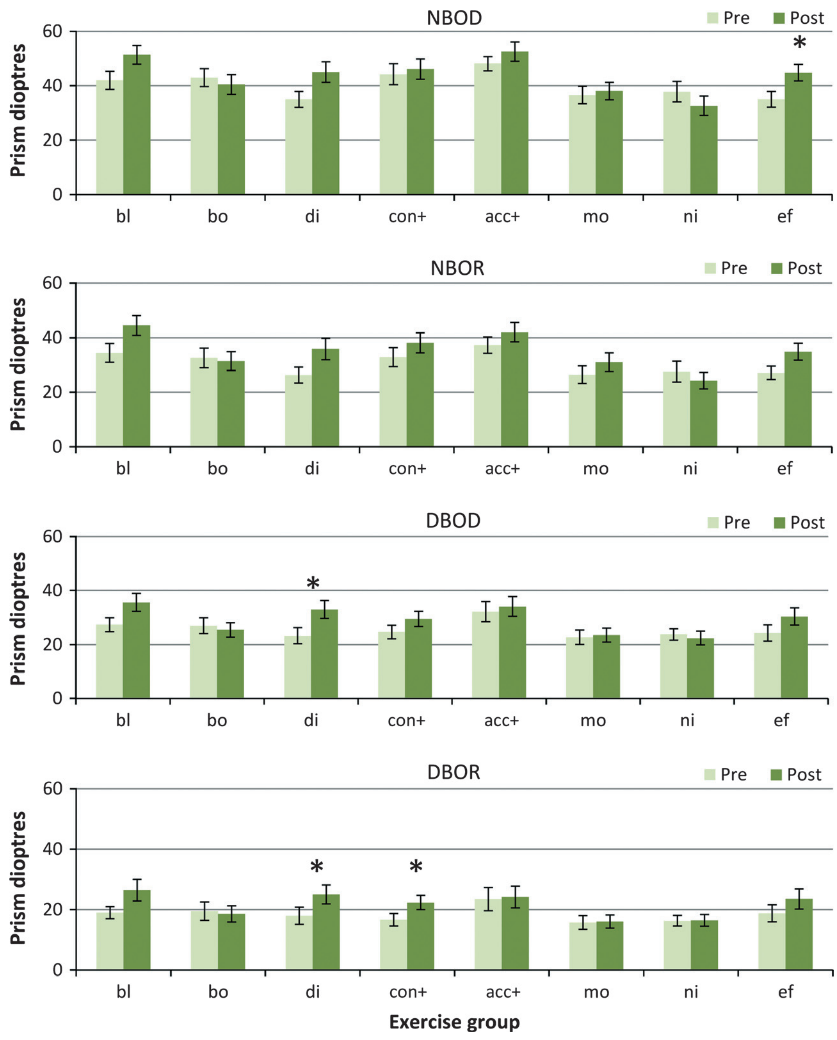

Figure 3. Base out fusion ranges. Pre- and post-treatment BO PFR measures for each treatment group. NBOD, near BO fusion range to diplopia; NBOR, near BO fusion recovery; DBOD, distance BO fusion range to diplopia; DBOR, distance base out fusion recovery. Group abbreviations; bl, Blur/accommodation treatment; bo, both (simultaneous convergence and accommodation treatment); di, disparity(convergence treatment); con+, convergence in excess of accommodation treatment; acc+, accommodation in excess of convergence treatment; mo, motion (placebo treatment); ni, nil (no treatment controls); ef, effort, no treatment. $*p < 0.01$.

alone also improved both near BI ranges marginally ($p = 0.05$ diplopia/$p = 0.03$ recovery).

Discussion

This study has helped identify how orthoptic exercises act, and goes some way to separate out true exercise effects from effort, practice and placebo effects. It has shown that in carefully controlled conditions, orthoptic exercises can induce medium term changes in clinical responses. Although it will not surprise experienced clinicians, it has also clearly shown that the additional influence of an enthusiastic therapist and the patient trying harder is a major factor independent of any practice or exercise modality. Placebo effects seem small, with few (and similar) changes in the Nil and Motion groups, confirming the sham exercises as a good placebo regime. We designed the study to mimic the experience of a patient being assessed before and after the start of treatment. We purposely did not assess repeatability of the tests because clinicians rarely do so. There was considerable between participant and test-retest variability in some measures which made smaller pre/post treatment changes statistically insignificant; particularly fusion range blur points which were so inconsistent that they were unusable. Treatment is commonly started after carrying out a test battery similar to ours, only doing each test once (or twice at most). This study has shown that eye exercises targeting a specific problem

© 2014 The Authors. Ophthalmic & Physiological Optics published by John Wiley & Sons Ltd on behalf of The College of Optometrists.

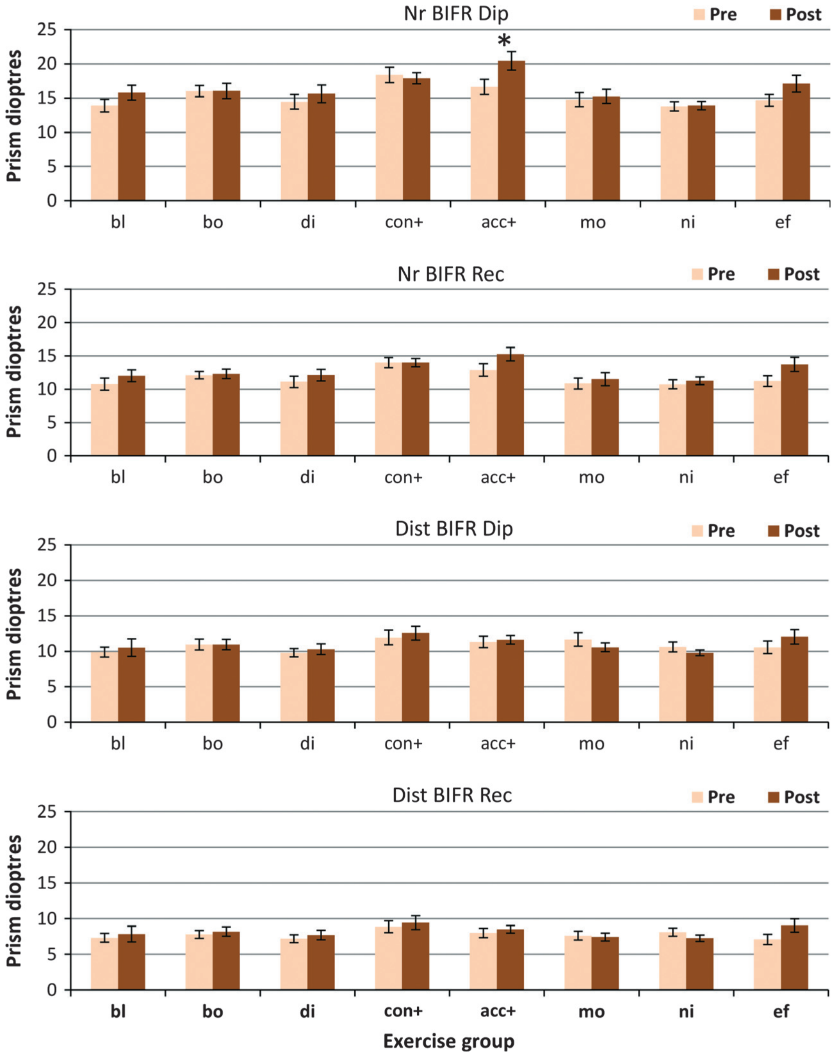

Figure 4. Base in fusion ranges. Pre- and post-treatment BI PFR measures for each treatment group. Nr BIFR Dip, near BI fusion range to diplopia; Nr BIFR Rec, near BI fusion recovery; Dist BIFR Dip, distance BI fusion range to diplopia; Dist BIFR Rec, distance BI fusion recovery. Group abbreviations; bl, blur/accommodation treatment; bo, both (simultaneous convergence and accommodation treatment); di, disparity (convergence treatment); con+, convergence in excess of accommodation treatment; acc+, accommodation in excess of convergence treatment; mo, motion (placebo treatment); ni, nil (no treatment controls); ef, effort, no treatment. (*),$p < 0.05$; *$p < 0.01$.

are not the only reason why response may improve on second testing, so practice, effort and placebo effects need to be considered.

We made great efforts to standardise testing methods, testing order and instructions at each visit. We also tried to ensure the level of effort required at home from each of the exercise groups was matched as closely as possible; each was given three exercises, to be practised for both near and distance in "jump" and gradual modalities. All were told to work hard at their exercises and to concentrate on achieving, and improving on, accurate and rapid responses. As in any study involving practice at home, it is possible that some participants told us they had practised, when they had not. We tried to mitigate this risk by stressing the importance of honesty in reporting. They were all science students and most were planning their own studies, which would be imprecise if their participants cheated. We also told them that we expected to find clear improvements in specific responses after specific exercises, so if they had not practised we would be able to tell – even though we were not sure the findings would be as clear as we implied! We asked them to fill in diary sheets which we subsequently checked. Although it was sometimes clear that the diary sheet had been filled in more assiduously by some than others, there appeared no systematic differences between the treatment groups.

© 2014 The Authors. Ophthalmic & Physiological Optics published by John Wiley & Sons Ltd on behalf of The College of Optometrists.

It is notable that all three regimes that exercised accommodation and convergence simultaneously (the Both, Con+ and Acc + groups) were in general less effective than in regimes that concentrated on one visual skill independent of the other. These "relative" vergence and accommodation exercises are a traditional mainstay of orthoptic exercises and vision therapy. Although changes in the predicted direction did occur when convergence or divergence were exercised in relation to accommodation e.g. near BI fusion range increased when divergence in relation to accommodation had been practiced, and distance BO fusion ranges increased when convergence had been practiced in relation to accommodation, these effects were often smaller than after practising accommodation or vergence separately. They may have been more difficult to alter in the relatively short timescale we used here, but, at least at the beginning of treatment, did not succeed in improving common measures significantly. We suggest that each element (response to blur, response to disparity, practice and particularly, effort) could act on both vergence and accommodation in an additive fashion during treatment to contribute to a better total outcome.

This study does have limitations. The main limitation is that we used typical, asymptomatic young adults, so findings may be different in patient groups; but we suggest that if we can find differences in typical groups, effects should only be larger in atypical groups where initial responses are further from ceiling, and exercises practiced for longer. Ceiling effects may have occurred for some of the measures such as near points of convergence and accommodation, but even so we did find significant improvements on these measures on the second visit. Some of the more "difficult" tests, such as VF and, particularly, monocular accommodation facility, seemed particularly prone to practice effects, improving significantly even in the control groups on second testing. Other measures seemed more responsive to exercise effects, such as near BO fusion ranges, while BI fusion ranges, especially in the distance, seemed relatively unresponsive. Further study would be necessary to investigate how exercises *plus* extra encouragement would act, and in patient groups with specific deficits rather than typical young adults.

Blur points during fusion range testing were a very unreliable measure, even in the academic young adults tested here, frequently not noticed or reported with so much delay that they were unusable in the analysis, even if their exercise regime had explicitly specified attention to blur. It is possible that our failure to find much improvement in the Con+ and Acc+ groups were partly because we could not reliably assess blur points, where differences might have been found, but we would also question the reliability of subjective blur points as a criterion.

This study looked mainly at subjective responses, relying on what the participants told us, and these subjective responses may be prone to differences in attention to details of blur or singularity of vision, and to differences in reaction times once thresholds are exceeded. Whether differences pre/post-treatment are reflected by *objective* change not subject to these effects occurs, and whether task-specific learning transfers into general vergence or accommodation tasks are only partly addressed by these findings. For example, practicing prism vergences is only useful if it changes vergence behaviour in the real world, and practicing clearing images through lenses is only useful if it changes accommodation for all close work: this is much more difficult to assess clinically.

We could have used dynamic retinoscopy as a more objective method of assessing accommodation,[24] with the participant fixating the target with one eye while refraction was assessed in the other, but chose not to for two reasons. In our laboratory we find such clear differences between objective accommodation measurements between monocular and binocular stimuli[9] that despite a significant literature describing dynamic retinoscopy[24–29] we remain concerned that changes in the quality of binocularity of the stimulus caused by the retinoscope flash might affect the accommodation. The other reason is that we were assessing accommodation objectively under truly monocular and binocular conditions in the other arm of the study.[19]

We acknowledge that we could have used different tests, testing order and instructions, and might have obtained different baseline results or more "accurate" responses e.g. by using additional minus lenses to test accommodation amplitude,[30] but by stressing the standard testing protocol and using within-subjects analysis, any such issues should be controlled as much as possible.

This is a complex dataset and the statistical analysis could have been carried out in many different ways. We acknowledge that multiple t-tests risk excessive Type I error (claiming significance when none exists), but carrying out conventional tests such as Bonferroni correction can be overly conservative[31] and in this case would not reflect the clinical changes that would be considered significant by clinicians. It is also arguable about how many comparisons should have been carried out e.g. between all eight groups or between placebo vs treatment, effort and no-extra-effort, between relative or yoked vergence and accommodation. By choosing an alpha level of 0.05, one in 20 significant t-tests would likely be false, but by choosing a more conservative alpha level of 0.01 this reduces to one in 100. We felt this represented the best compromise and represented clinically significant differences.

Our findings are broadly in line with those of Maxwell *et al.*[8] who suggested a strong role for vergence accommo-

 © 2014 The Authors. Ophthalmic & Physiological Optics published by John Wiley & Sons Ltd on behalf of The College of Optometrists.

dation and volitional vergence in vergence and accommodation facility. The influence of accommodative convergence is often stressed by clinicians, but although in this study we found that practicing accommodation (the Blur group) helped some aspects of convergence such as near point of convergence and BO fusion range, there were no corresponding *objective* changes, particularly in accommodation found in the other arm of the study.[19] It is therefore not clear how improvements of clinical test results translate to everyday focusing.

Participants in the Effort group exhibited a greater number of improvements in response and these improvements were larger. Accommodation in particular seems particularly altered by effort and instructions, as well as the known factors such as age and demand discussed by Wick *et al.*[15] Many of these asymptomatic participants only reported blur, or bothered to clear images, if they are strongly encouraged to do so. This, and the fact that so few people noticed blur points when the fusion range was tested, may suggest that blur is often tolerated and unnoticed unless attention is specifically directed to it.

Monocular accommodative facility seemed particularly sensitive to practice effects; it improved in all but one group (even both the control groups), and only failed to improve if allowing blur had been particularly specified during practice. It particularly improved when it had been one of the homework tasks (in the Blur group), but this did not particularly transfer to even binocular accommodative facility.

The main conclusions are first, that instruction set and levels of effort required and exerted on the part of both therapist and patient are a major factor in improvements that occur in clinical responses. Enthusiastic, encouraging therapists and just trying harder seem to be vital to success and the type of exercise itself is less critical. We accept it is quite likely all these effects may be additive. Clinicians must be aware of this is and be very careful of claims about the effect of any specific exercise. If a particular treatment is to be tested, levels of instruction, effort and reward must be carefully standardised before and after treatment.

Secondly exercising "relative vergences" seems much less effective, or possibly much slower to take effect, than exercising accommodation, and particularly convergence, independently of each other. We suggest that pure accommodation or convergence exercises have a more immediate effect and act with extra effort in an additive fashion to provide a range of alternative routes that individuals drive better near responses.

Disclosures

The authors report no conflicts of interest and have no proprietary interest in any of the materials mentioned in this article.

Acknowledgements

AH and ST are supported by a UK Medical Research Council Clinician Scientist Fellowship (G0802809) awarded to AH.

References

1. Barrett B. A critical evaluation of the evidence supporting the practice of behavioural vision therapy. *Ophthalmic Physiol Opt* 2009; 29: 4–25.
2. Convergence Insufficiency Treatment Study Group. Randomized clinical trial of treatments for symptomatic convergence insufficiency in children. *Arch Ophthalmol* 2008; 126: 1336–1349.
3. Convergence Insufficiency Treatment Study Group. Long-term effectiveness of treatments for symptomatic convergence insufficiency in children. *Optom Vis Sci* 2009; 86: 1096–1103.
4. Griffin J & Grisham J. Binocular Anomalies: Diagnosis and Vision Therapy. Butterworth-Heinemann: Boston, 2002.
5. Scheiman M & Wick B. Clinical Management of Binocular Vision. Lippincott Williams & Wilkins: Philadelphia, 2008.
6. Ansons A & Davis H. Diagnosis and Management of Ocular Motility Disorders. Blackwell Science: Oxford, 2001.
7. Alvarez TL & Kim EH. Analysis of saccades and peak velocity to symmetrical convergence stimuli: binocularly normal controls compared to convergence insufficiency patients. *Invest Ophthalmol Vis Sci* 2013; 54: 4122–4135.
8. Maxwell J, Tong J & Schor CM. Short-term adaptation of accommodation, accommodative vergence and disparity vergence facility. *Vision Res* 2012; 62: 93–101.
9. Horwood A & Riddell P. The use of cues to convergence and accommodation in naïve, uninstructed participants. *Vision Res* 2008; 48: 1613–1624.
10. Judge S. How is binocularity maintained during convergence and divergence? *Eye (Lond)* 1996; 10: 172–176.
11. Caltrider N & Jampolsky A. Overcorrecting minus lens therapy for treatment of intermittent exotropia. *Ophthalmology* 1983; 90: 1160–1165.
12. Kushner BJ. Does overcorrecting minus lens therapy for intermittent exotropia cause myopia? *Arch Ophthalmol* 1999; 117: 638–642.
13. Rouse MW, Deland PN, Chous R & Determan TF. Monocular accommodative facility testing reliability. *Optom Vis Sci* 1989; 66: 72–77.
14. Rouse MW, DeLand PN, Mozayani S & Smith JP. Binocular accommodative facility testing reliability. *Optom Vis Sci* 1992; 69: 314–319.
15. Wick B, Yothers TL, Jiang BC & Morse SE. Clinical testing of accommodative facility: part 1. A critical appraisal of the literature. *Optometry* 2002; 73: 11–23.
16. Ciuffreda K & Hokoda S. Effect of instruction set and higher level control on the accommodative response spatial frequency profile. *Ophthalmic Physiol Opt* 1985; 5: 221–223.

© 2014 The Authors. Ophthalmic & Physiological Optics published by John Wiley & Sons Ltd on behalf of The College of Optometrists.

17. Stark LR & Atchison DA. Subject instructions and methods of target presentation in accommodation research. *Invest Ophthalmol Vis Sci* 1994; 35: 528–537.
18. Ciuffreda K, Rosenfield M, Rosen J, Azimi A & Ong E. Accommodative responses to naturalistic stimuli. *Ophthalmic Physiol Opt* 1990; 10: 168–174.
19. Horwood A, Toor S & Riddell P. Change in convergence and accommodation after two weeks of eye exercises in typical young adults. *J AAPOS* 2014. (in press)
20. Convergence Insufficiency Treatment Study Group. The convergence insufficiency treatment trial: design, methods, and baseline data. *Ophthalmic Epidemiol* 2008; 15: 24–36.
21. Rouse M, Borsting E, Mitchell GL *et al.* Validity of the convergence insufficiency symptom survey: a confirmatory study. *Optom Vis Sci* 2009; 86: 357–363.
22. Rosenfield M, Ciuffreda KJ, Ong E & Super S. Vergence adaptation and the order of clinical vergence range testing. *Optom Vis Sci* 1995; 72: 219–223.
23. Bharadwaj S & Candy T. Cues for the control of ocular accommodation and vergence during postnatal human development. *J Vis* 2008; 8: 1–16.
24. Alvarez TL, Kim EH, Vicci VR *et al.* Concurrent vision dysfunctions in convergence insufficiency with traumatic brain injury. *Optom Vis Sci* 2012; 89: 1740–1751.
25. Brookman KE. A retinoscopic method of assessing accommodative performance of young human infants. *J Am Optom Assoc* 1981; 52: 865–869.
26. Hunter DG. Dynamic retinoscopy: the missing data. *Surv Ophthalmol* 2001; 46: 269–274.
27. McClelland J & Saunders K. The repeatability and validity of dynamic retinoscopy in assessing the accommodative response. *Ophthalmic Physiol Opt* 2003; 23: 243–250.
28. Tarczy-Hornoch K. Modified Bell retinoscopy: measuring accommodative lag in children. *Optom Vis Sci* 2009; 86: 1337–1345.
29. Woodhouse JM, Meades JS, Leat SJ & Saunders KJ. Reduced accommodation in children with Down syndrome. *Invest Ophthalmol Vis Sci* 1993; 34: 2382–2387.
30. Atchison DA, Capper EJ & McCabe KL. Critical subjective measurement of amplitude of accommodation. *Optom Vis Sci* 1994; 71: 699–706.
31. Perneger T. What's wrong with Bonferroni adjustments. *BMJ* 1998; 316: 1236–1238.

Supporting Information

Additional Supporting Information may be found in the online version of this article:

Data S1. Testing protocol.

© 2014 The Authors. Ophthalmic & Physiological Optics published by John Wiley & Sons Ltd on behalf of The College of Optometrists.
